# Vision-Threatening Behcet's Disease: Severity of Ocular Involvement Predictors

**DOI:** 10.1155/2018/9518065

**Published:** 2018-04-26

**Authors:** Mohammed A. Hussein, Iman M. Eissa, Ahmed A. Dahab

**Affiliations:** ^1^Internal Medicine Department, Rheumatology and Clinical Immunology Division, Cairo University, Cairo, Egypt; ^2^Ophthalmology Department, Cairo University, Cairo, Egypt

## Abstract

**Purpose:**

To examine and spot systemic findings commonly associated with a serious form of ocular Behcet's disease. This could potentially help ophthalmologists categorize their patients based on future risk and plan treatment accordingly.

**Subjects and Methods:**

The data of 249 patients with Behcet's disease were examined thoroughly. Correlations between systemic and ocular findings were recorded. Patients were further subgrouped by the authors as having a vision-threatening form of the disease or not. Regression analysis was done to spot predictors for a vision-threatening form of the disease.

**Results:**

The presence of systemic vasculitis and oral and genital ulcers in a patient with Behcet's disease was found to be associated with a milder form of ocular affection or none at all and vice versa. Certain correlations between findings were also found.

**Conclusion:**

Certain findings in Behcet's disease may act as predictors for the severity of ocular affection. Directing our attention to these factors by the internist and ophthalmologist can help plan the frequency of follow-up as well as the aggressiveness of treatment in patients with Behcet's disease.

## 1. Introduction

Behcet's disease (BD) is a chronic, relapsing, inflammatory vascular disease of unknown etiology that commonly affects the eye with unpredictable exacerbations and remissions. Various visual complications may occur and are potentially sight threatening [1]. Male sex and a younger age of disease onset (younger than 40 years) were found to be associated with a higher frequency of ocular affection as well as of more severe form of the disease [1]. To date however, there are no gold standard criteria for the diagnosis/classification of Behcet's disease, with more than 15 available sets of criteria used around the world [2]. Two of the most commonly used classification criteria are the ICBD (International Criteria for Behcet's Disease) which was used in this study and the ISG (International Study Group) criteria. Egypt is one of the countries with a high incidence of Behcet's disease. A 4-year follow-up study done on Egyptian patients with Behcet's disease demonstrated a higher male-to-female ratio and a higher incidence of neurological (34.9%) and vascular (57.1%) lesions than other countries. This study also demonstrated a 47.6% ocular affection of varying degrees among its 63 studied patients [3]. Another study done in Cairo University, Egypt, found that the ICBD criteria perform better on Egyptian patients more than other available classifications [4]. Performance of the ISG, ICBD 2006, revised Japanese criteria, and the revised ICBD 2010 was evaluated in terms of sensitivity, specificity, negative likelihood ratio (NLR), negative predictive value (NPV), positive predictive value (PPV), positive likelihood ratio (PLR), diagnostic odds ratio (DOR), and Youden's index (YI), and it was found that the ICBD 2010 carried the highest sensitivity (98.83%), NPV (98.48%), DOR (1645), and YI (0.94) and the lowest NLR (0.01) [4].

The common ocular manifestations of Behcet's disease which potentially threaten vision include anterior and posterior uveitis, vitritis, panuveitis, retinal vasculitis, papillitis, and chorioretinitis. Complications like retinal detachment, secondary glaucoma, and optic atrophy often cause irreversible visual loss. It is thus very important for an ophthalmologist to be able to predict which case of Behcet's disease will pursue a vision-threatening course and which case will not. To date, no enough data exists regarding the presence of general predictors for vision-threatening disease. If vision-threatening disease has certain predictors (clinical as well as demographic), detecting these predictors early in the course of the disease may help alter the course of the disease in these high-risk patients. A patient with a potentially vision-threatening form of the disease can be given special ophthalmological care, closer follow-up appointments, more aggressive treatment, and/or earlier intervention.

The authors further pursued this point of research in an attempt to look for clinical predictors for the potentially vision-threatening form of Behcet's disease. In the hope that this can allow ophthalmologists and internists to take appropriate measures in a timely fashion, it was for this purpose that this work was done.

## 2. Subjects and Methods

The study was done in accordance with the ethical standards of the national research committee and the 1964 Helsinki Declaration and its later amendments [5].

This retrospective analytical database study targeted patients attending the outpatient clinic of Internal Medicine, Rheumatology and Clinical Immunology Division of Kasr Al Ainy Medical School, Cairo University. The data of this historical cohort were collected from the available fully completed and revised files of 249 Egyptian patients with Behcet's disease recorded from the end of 2012 to the end of 2017.

The revised International Criteria for Behcet's Disease (ICBD) 2010 [6] was chosen to confirm patients to have the disease according to a recent work by Hussein et al. [4]. These criteria include the following:

- Recurrent oral ulcers (OU) of at least 3 times/year
- Recurrent genital ulcers (GU)
- Cutaneous manifestations including papulopustular rash and erythema nodosum
- Ocular manifestations consistent with the disease as uveitis (anterior, posterior, or panuveitis) and retinal vasculitis, chorioretinitis, or papillitis
- Vascular manifestations including venous thromboembolism, superficial thrombophlebitis, arterial thrombosis, and aneurysms, especially aortic and pulmonary aneurysms
- Central nervous system (CNS) lesions consistent with the disease, namely, parenchymal CNS involvement and/or venous sinus thrombosis
- Pathergy test as an extra criterion to be used if conducted and positive

A weighted point value system was given: two points for oral ulcers (OU), genital ulcers (GU), and ocular manifestations and only one point for the others, and at least four points are required for diagnosing the patient as having the disease. Patients who had other autoimmune and/or autoinflammatory diseases were excluded from the study. All patients had a duration of illness of at least 4 years and were treated by corticosteroids and methotrexate.

Ophthalmologically, patients' data was thoroughly examined and the cohort was divided by the authors into two groups: a group with a “vision-threatening form of Behcet's disease” and another group with “non-vision-threatening Behcet's disease.” The full ophthalmological examination done at the ophthalmology department outpatient clinics included anterior segment examination by slit lamp biomicroscopy, posterior segment examination by 90-diopter lens, and indirect ophthalmoscopy. Fundus fluorescein angiography was done if needed to confirm posterior segment findings like retinal vasculitis. Visual acuity was measured as well as intraocular pressure by applanation tonometry for each patient. Patients with a best-corrected visual acuity (BCVA) of 6/60 or less due to an irreversible cause, as well as those with documented panuveitis, retinal vasculitis, chorioretinitis, and/or papillitis or a combination of these findings, were classified by the authors as having a vision-threatening form of the disease (VTD), while patients whose BCVA was 6/36 or better, those who had a reversible cause of visual loss, or those who were found to have mild-to-moderate anterior uveitis, vitritis (diffuse vitreous cells and/or mild-to-moderate vitreous haze), complicated cataract (which can be surgically removed), episcleritis, and/or a combination of these were classified as having a non-vision-threatening form of the disease (NVTD).

Each ocular finding (like the presence of anterior or posterior uveitis, panuveitis, vitritis, retinal vasculitis, chorioretinitis, papillitis, and ocular complications like cataract, glaucoma, macular edema, and retinal detachment) was recorded if present for each included patient. Each systemic finding (like the presence of oral ulcers, genital ulcers, cutaneous manifestations, a positive pathergy test, systemic vasculitis, CNS manifestations, arthralgia, arthritis, and/or gastrointestinal tract (GIT) symptoms) was also recorded if present for each participating patient. Age, sex, laterality of ocular disease, and any family history for the disease were also recorded for each patient.

Data were statistically described in terms of mean ± standard deviation (SD), range, or frequencies (number of cases) and percentages when appropriate. Comparison between the study groups was done using the chi-square (*χ*^2^) test. An exact test was used instead when the expected frequency was less than 5. Correlation between various variables was done using Spearman's rank correlation equation. Multivariate logistic regression analysis was used to test for the preferential effect of all important variables on the occurrence of vision-threatening affection. A *p* value of less than 0.05 was considered statistically significant. All statistical calculations were done using the computer program IBM SPSS (Statistical Package for the Social Science; IBM Corp., Armonk, NY, USA) release 22 for Microsoft Windows.

## 3. Results

### 3.1. Descriptive Statistics

The records of 249 patients were included in our study. The mean age in our study was 32.3 ± 8.47 years (mean ± SD). 85.1% of our patients were males (212 patients), and 14.9% (37 patients) were female (yielding a male-to-female ratio of 5.7 : 1). Of these, 38.2% (95 patients) had bilateral ocular affection. A positive family history was only found in 3.2% of patients (8 patients).

As for overall ocular affection, a total of 51% (127 patients) of all patients were found to have some form of ocular involvement by the disease.

Of these, 59% suffered from anterior uveitis, 74.8% from posterior uveitis, and 33.8% from panuveitis. 54.3% of these patients had retinitis, 46.4% had vitritis, 66.9% had chorioretinitis, 31.4% had retinal vasculitis, 7.8% had papillitis, 9.4% had macular edema, and 7.8% had secondary retinal detachment. Patients frequently suffered from more than one ocular finding.

According to the authors' suggested classification previously described, 74.01% of these patients (94 patients) were found to fall within the category of having vision-threatening disease (decreased vision due to an irreversible cause), while 25.98% (33 patients) fell in the category of having non-vision-threatening disease.

Systemically, 98.8% of all patients suffered from oral ulceration, 90% had genital ulceration, 36.5% had different skin manifestations of Behcet's disease, 34.5% suffered from arthralgia, 31.3% had systemic vasculitis, 17.7% manifested a positive pathergy test, 12% had CNS manifestations, 10% had arthritis, and 2.8% had different GIT manifestations of Behcet's disease, with most patients displaying more than one systemic finding.

#### 3.1.1. Correlation between Individual Ocular and Systemic Variables

Upon correlating all variables with each other, many correlations were found. Some were statistically significant, and some showed only a trend towards being significant.  Table 1 displays these correlations and their correlation coefficient (*r*) and *p* value. It was generally noted that genitourinary ulcers as well as peripheral vasculitis almost always had a significant negative correlation with all forms of ocular affection whether uveitis, vitritis, chorioretinitis, or retinal vasculitis. Oral ulcers were also inversely correlated with the presence of retinal vasculitis, as well as with overall ocular involvement. However, this correlation was only close to being statistically significant (Table 1).

Secondary glaucoma and complicated cataract were both significantly positively correlated with the presence of GIT symptoms, and secondary retinal detachment was significantly positively correlated with the presence of a positive pathergy test.

#### 3.1.2. Correlation between Different Variables and the Occurrence of Vision-Threatening Disease (VTD)

Upon correlating systemic variables with the development of the vision-threatening form of ocular affection (as previously described), it was found that 65.2% of participants with genitourinary ulcerations did not develop vision-threatening disease, whereas 34.8% with genitourinary symptoms had vision-threatening disease. 64% of patients with no genitourinary affection developed VTD. This relationship was statistically highly significant (*p* = 0.008).

Patients with systemic vasculitis largely developed non-vision-threatening disease, as 79.5% of them exhibited a mild form of ocular affection as opposed to 20.5% of patients with systemic vasculitis who exhibited VTD. This again was statistically highly significant (*p* = 0.001).

More than 50% of patients with oral ulcers developed non-vision-threatening disease. Meanwhile, 66.7% of patients without oral ulceration (2 out of 3 patients) developed VTD. The difference was however statistically nonsignificant (*p* = 0.55) and of limited clinical significance due to the small number of patients having no oral ulcers (3 patients).

The rest of systemic variables, as well as family history (*p* = 0.26), had no significant effect on the development of VTD. It seems that the presence of genital ulcers and systemic vasculitis may have a significant protective role against the development of the vision-threatening form of the disease.

#### 3.1.3. Logistic Regression and Predictive Factors

Upon logistic regression analysis, taking into consideration each variable alone (Table 2), the authors found a significant predictive value for both the presence of genital ulcers (*p* = 0.004) and that of systemic vasculitis (*p* = 0.001) on the development of NVTD.

Upon logistic regression analysis with all variables taken into consideration (Table 3), the authors found that three factors have a predictive value on the development of the non-vision-threatening form of the disease. Namely, these are the presence of genital ulcers (*p* = 0.003) and systemic vasculitis (*p* = 0.001) which both showed a highly significant predictive value on the development of non-vision-threatening disease. The presence of oral ulcers with a *p* value close to being significant (*p* = 0.07) may have a predictive value as to the development of NVTD, yet still it is statistically nonsignificant. In other words, absence of these systemic criteria (GU, systemic vasculitis, and maybe OU as well) may put the eye at higher risk for the development of vision-threatening disease (VTD).

## 4. Discussion

Behcet's disease (BD) is named after Turkish dermatologist Hulusi Behçet who reported three cases with recurrent oral and genital ulceration together with hypopyon anterior uveitis [7]. It is a chronic relapsing multisystem disease of yet nonconclusive etiology. A complex causality involving infectious agent exposure as well as genetic and environmental factors may play a role [8]. A previous study done on 63 Egyptian patients with BD studying their demographics found that a very high male-to-female ratio was exhibited (30.5 : 1) as well as a mean age of 32.8 ± 8.3 years [3]. The commonest manifestation of BD in this study was oral ulcers (100%), followed by genital ulcers (96.8%), vascular lesions (57.1%), cutaneous manifestations (55.5%), ocular affection (47.6%), joint affection (36.5%), CNS (34.9%), gastrointestinal manifestations (19%), and lastly cardiac manifestations (6.3%) [3]. Despite the difference in sample size (249 versus 63 subjects), the reported percentages were comparable to ours except for CNS, GIT, and cardiac manifestations. We also reported a different male-to-female ratio (5.7 : 1) which was lower than the ratio of this study but higher than the ratio of most studies done in the Mediterranean region.

Another study on the demographics of BD in the North African Mediterranean region (Tunisia) implementing a closer sample size to ours (260) showed a male-to-female ratio of 2.6 : 1, oral ulcers in 100% of patients, genital ulcers in 83%, ocular involvement in 51% (the same percentage as ours), arthritis in 38.8%, vasculitic and thrombotic lesions in 33%, neurological lesions in 24.2%, and GIT symptoms in 1.5%. These results are almost identical to what we found in our study. This study also reported a high overall incidence of DVT and a low incidence of GIT symptoms which were similar to our results [9].

We found that different correlations exist between different variables. Genital ulcers were almost always negatively correlated with all sorts of ocular inflammation, and so was systemic vasculitis. A significant negative correlation existed between these two variables and the development of anterior or posterior uveitis, vitritis, retinitis, chorioretinitis, panuveitis, and overall ocular involvement. This finding was similar to that of the previous study where the authors reported less frequent occurrence of deep vein thrombosis as well as GU in patients with ocular involvement [9].

We found a predictive potential for the presence of GU, systemic vasculitis, and OU on the development of what we classified as non-vision-threatening disease, where we reported a milder form of eye affection in the presence of one or more of these three systemic criteria. Interestingly, a previous study by Hazirolan et al. found that GU were significantly lower among BD patients with ocular (71.4%) compared to those without ocular affection (*p* = 0.04), but the authors did not propose any explanation for this in their study [10]. To date, no studies have proposed evidence-based explanation for this phenomenon of what we call “immunological targeting” and why severe inflammation of ocular structures is usually associated with a milder peripheral inflammatory process and vice versa “reverse immunological targeting.” A lot of research can be started to fully explain this phenomenon. However, we postulate that retinal pericytes, which may play a role in intravascular immunity, are derived from the same embryologic origin as CNS pericytes and thus may behave differently from peripheral vascular pericytes [11]. The eye as an embryologic outgrowth from the brain may be immunologically protected, so that in the setting of a florid form of peripheral vasculitis, orogenital ulcers, and mucocutaneous manifestations, the eye may still be relatively protected. It is however still not understood why absence of these peripheral findings is usually associated with the vision-threatening form of ocular inflammation. Further investigation is thus needed to investigate this finding shedding more light on the possibility of having two immunological variants of Behcet's disease, one with a central impact (more severe ocular affection) and the other with a peripheral one (more severe systemic vasculitis). HLA subtypes of the disease may also contribute to the type of systemic involvement, a point which needs further study and is somehow a limitation to our study.

Besides, the immune privilege (IP) manifested by ocular and brain tissue in particular and described as a suppressed or extremely extinguished immune response to foreign antigens, particularly alloantigens, functions normally as a homeostatic mechanism preserving function in highly specialized tissues with a limited capacity for renewal such as those of the brain and eye [12]. However, IP (in the form of blood-ocular barriers, induction of T regulatory cells, intraocular immune modulators, lack of lymphatics, and other properties) is relatively easily bypassed when facing a sufficiently strong immunological response. Under these circumstances, these privileged tissues (those of the eye and brain) may be at greater risk of collateral damage because the natural defence mechanisms of these privileged tissues are more easily breached than those of a fully immunocompetent tissue which quickly rejects foreign antigens and restores integrity [13]. This acts like a double-edged knife to an organ like the eye. Under most circumstances, immune privilege (IP) mechanisms maintain tissue integrity; however, when these mechanisms are breached, various degrees of tissue damage occur from severe tissue destruction in retinal viral infections and other forms of uveoretinal inflammation. Immune privilege does not appear to offer much protection against the damaging effects of uveitis possibly because IP serves mainly to maintain homeostasis, mainly keeping healthy tissue free of random antigens which may provoke inflammation. However, when facing a serious challenge, IP drearily fails to prevent severe inflammation. In uncontrolled vision-threatening uveitis, both infection and the immune response to it can cause permanent structural damage. Therapies such as anti-TNF*α* are claimed to impair the destructive effects of inflammation while permitting monocytes to traverse tissues without causing damage [14]. This whole IP mechanism may partially explain the exaggerated ocular immune response to Behcet's disease in certain cases that the authors postulate. However, it does not fully explain the opposite scenario where in the presence of marked ocular inflammation, a quiet systemic (peripheral) pattern of disease usually coexists. This is a limitation to our study that needs further research.

In our study, we noted a positive correlation between bilaterality of ocular affection and the presence of arthralgia, the presence of vitritis and positive pathergy, anterior uveitis and arthralgia, retinal vasculitis and positive pathergy, retinal vasculitis and increasing age, GIT symptoms and the development of glaucoma and cataract (this specific correlation may be attributed to complications of prolonged use of corticosteroids), and finally retinal detachment and positive pathergy. All these correlations—except the last three—were close to being statistically significant. Whether immunologic variants or HLA subtypes of the disease exist that could be responsible for this “symptom pairing” needs to be further investigated. HLA subtypes of the disease may also contribute to the type of systemic involvement, a point which needs further study and is somehow a limitation to our study.

Most vision-threatening conditions were of posterior segment origin and were largely inversely correlated with the presence of genital ulcers and systemic vasculitis. This agrees with what another study reported, in which posterior segment lesions in Behcet's disease are of a persistent nature and lead to significant visual loss [15]. However, this study did not shed any light on the strong negative correlation we reported between the severity of posterior segment affection and certain systemic features.

Behcet's disease remains an immunologic mystery, a clinical and a therapeutic challenge to internists, immunologists, and ophthalmologists alike. The importance of long-term follow-up remains crucial in the prognosis of the disease. Potential benefits of long-term colchicine treatment were previously discussed and showed some promising results [16]. With further collaborative research and understanding of the disease and its patterns of organ affection and/or avoidance, we may reach new classifications which will help us better plan our treatment and follow-up regimens and even develop prophylactic options. We recommend that predictors for severity of ocular diseases mentioned in this study be implemented in the initial assessment for disease and its diagnostic criteria and eventually be taken into consideration when designing the treatment plan.

## Figures and Tables

**Table 1:** Correlation between individual variables.

Correlated variables	Correlation coefficient (*r*)	*p* value
Bilaterality & GU	−0.15	0.018^∗^
Bilaterality & systemic vasculitis	−0.21	0.001^∗^
Bilaterality & arthralgia	0.108	0.09
Anterior uveitis & systemic vasculitis	−0.198	0.002^∗^
Anterior uveitis & CNS	−0.109	0.08
Anterior uveitis & arthralgia	0.112	0.07
Anterior uveitis & male gender	−0.119	0.06
Posterior uveitis & GU	−0.178	0.005^∗^
Posterior uveitis & systemic vasculitis	−0.245	0.001^∗^
Retinitis & systemic vasculitis	−0.186	0.003^∗^
Vitritis & GU	−0.191	0.002^∗^
Vitritis & pathergy	0.113	0.07
Vitritis & systemic vasculitis	−0.193	0.002^∗^
Vitritis & CNS	−0.14	0.019^∗^
Chorioretinitis & GU	−0.12	0.047^∗^
Chorioretinitis & systemic vasculitis	−0.26	0.001^∗^
Chorioretinitis & CNS	−0.13	0.031^∗^
Retinal vasculitis & OU	−0.15	0.016^∗^
Retinal vasculitis & GU	−0.109	0.08
Retinal vasculitis & pathergy	0.11	0.07
Retinal vasculitis & systemic vasculitis	−0.13	0.04^∗^
Retinal vasculitis & age	0.11	0.06
Panuveitis & systemic vasculitis	−0.19	0.002^∗^
Panuveitis & male gender	−0.138	0.03^∗^
Ocular involvement & OU	−0.1	0.08
Ocular involvement & GU	−0.22	0.001^∗^
Ocular involvement & systemic vasculitis	−0.27	0.001^∗^
Ocular involvement & CNS	−0.13	0.03^∗^
Glaucoma & GIT	0.227	0.001^∗^
Cataract & GIT	0.149	0.019^∗^
Synechia & male gender	−0.13	0.03^∗^
Retinal detachment & pathergy	0.17	0.006^∗^

^∗^Statistically significant.

**Table 2:** Univariate regression analysis and significance of each variable.

Variables	Score	df	*p* value
OU	1.080	1	0.299
GU	8.148	1	0.004^∗^
Cutaneous	0.516	1	0.472
Pathergy	0.671	1	0.413
Systemic vasculitis	14.362	1	0.001^∗^
CNS	1.783	1	0.182
Arthralgia	0.944	1	0.331
Arthritis	0.391	1	0.532
GIT	1.153	1	0.283
Family history	2.243	1	0.134
Age	0.157	1	0.692
Male gender	0.000	1	0.991

^∗^Statistically significant.

**Table 3:** Multivariate regression analysis with all variables in the equation.

Variables	*B*	SE	df	*p* value
OU	−2.29	1.29	1	0.077^∗^
GU	−1.45	0.48	1	0.003^∗^
Cutaneous	0.32	0.30	1	0.294
Pathergy	0.38	0.37	1	0.311
Systemic vasculitis	−1.37	0.35	1	0.001^∗^
CNS	−0.46	0.47	1	0.328
Arthralgia	0.18	0.30	1	0.543
Arthritis	−0.28	0.50	1	0.574
GIT	1.25	0.86	1	0.146
Family history	−1.40	1.11	1	0.208
Age	−0.01	0.01	1	0.452
Male gender	0.23	0.40	1	0.561

^∗^Statistically significant.
